# A modeling and machine learning approach to ECG feature engineering for the detection of ischemia using pseudo-ECG

**DOI:** 10.1371/journal.pone.0220294

**Published:** 2019-08-12

**Authors:** Carlos A. Ledezma, Xin Zhou, Blanca Rodríguez, P. J. Tan, Vanessa Díaz-Zuccarini

**Affiliations:** 1 Department of Mechanical Engineering, University College London, London, United Kingdom; 2 Department of Computer Science, University of Oxford, Oxford, United Kingdom; 3 Wellcome/EPSRC Centre for Interventional and Surgical Sciences (WEISS), Department of Medical Physics and Biomedical Engineering, University College London, W1W 7TS, UK; University of Minnesota, UNITED STATES

## Abstract

Early detection of coronary heart disease (CHD) has the potential to prevent the millions of deaths that this disease causes worldwide every year. However, there exist few automatic methods to detect CHD at an early stage. A challenge in the development of these methods is the absence of relevant datasets for their training and validation. Here, the ten Tusscher-Panfilov 2006 model and the O’Hara-Rudy model for human myocytes were used to create two populations of models that were in concordance with data obtained from healthy individuals (control populations) and included inter-subject variability. The effects of ischemia were subsequently included in the control populations to simulate the effects of mild and severe ischemic events on single cells, full ischemic cables of cells and cables of cells with various sizes of ischemic regions. Action potential and pseudo-ECG biomarkers were measured to assess how the evolution of ischemia could be quantified. Finally, two neural network classifiers were trained to identify the different degrees of ischemia using the pseudo-ECG biomarkers. The control populations showed action potential and pseudo-ECG biomarkers within the physiological ranges and the trends in the biomarkers commonly identified in ischemic patients were observed in the ischemic populations. On the one hand, inter-subject variability in the ischemic pseudo-ECGs precluded the detection and classification of early ischemic events using any single biomarker. On the other hand, the neural networks showed sensitivity and positive predictive value above 95%. Additionally, the neural networks revealed that the biomarkers that were relevant for the detection of ischemia were different from those relevant for its classification. This work showed that a computational approach could be used, when data is scarce, to validate proof-of-concept machine learning methods to detect ischemic events.

## Introduction

Coronary heart disease (CHD) is an occlusion of the vessels that irrigate the heart. Even though sometimes CHD is asymptomatic, it is very likely to cause ischemia and infarction. Current clinical guidelines suggest to target personalized therapies to patients at high risk [1] to reduce the deaths by CHD, but there exist few automatic methods for individual risk assessment. Consequently, clinicians rely on visual ECG inspection, which has been proven to be affected by personal bias and fatigue [2]. Furthermore, inter-subject variability plays a defining role when assessing this risk: a healthy-looking patient can suddenly die of CHD and a patient considered at high risk can live many years without showing indications of the underlying pathology. Moreover, CHD often presents itself as a ‘silent’ disease, showing no symptoms, and a heart attack or a stroke is the first warning [3]. Consequently, assessing the individual risk of suffering from coronary heart disease is today one of the most pressing issues in biomedical engineering.

The study of the effects of ischemia *in-vivo* in humans is often affected by many methodological factors [4] and is limited by small cohorts of patients because of the invasiveness of the necessary acquisitions and scarcity of suitable individuals [5, 6]. Indeed, there are very few public electrocardiogram (ECG) recordings databases specifically acquired for the study of ischemia, and the few that exist contain a limiting number of caveats. For instance, the STAFFIII database [7–9], one of the most relevant ECG databases for the study of ischemia to date, contains just 104 patients, aged 60.77 ± 11.57 years, with pre-existing cardiac conditions and varied morphological features (e.g. location of myocardial infarction or which artery was occluded); these factors, commonly found in clinical acquisitions concerning ischemia, compromise the clinical extrapolation of the findings of ischemia studies in humans [4]. Moreover, the lack of relevant datasets has precluded the development of machine learning methods for the detection of early-stage ischemic events because they fail to generalize. Indeed, machine learning techniques, in the context of ECG analysis, have mostly been developed for the detection of abnormal cardiac rhythms, as can be consulted in the recent review by Lyon et al. [10], or for the detection and localization of myocardial infarction, as has been summarized by Acharya et al. [11]; to the best of our knowledge, there is no machine learning technique applied to the early detection of ischemia that has successfully generalized to clinical practice. However, previous research has already highlighted that computer simulations could be used to generate large datasets suitable for the training of machine learning algorithms [10]. This paper was motivated by the hypothesis that a computational study, *prior* to a clinical trial, could be beneficial to the development of ischemia-detection methods.

Recent studies have investigated the effects of ischemia through mathematical models of cardiac electrophysiology (EP) [12–15]. The introduction of ischemia in computational models has allowed the study of its effects in single cells, cables of cells, slabs of tissue and whole heart models. Some whole-heart *in-silico* studies have also calculated and analyzed ECG signals from ischemic hearts [16–18]. An advantage of these computational studies, over their experimental counterparts, is that the severity and size of the ischemic event can be modulated in detail. Moreover, computational simplifications (i.e. 1D cable of cells or 2D slab of tissue instead of 3D heart geometry) already enable the study of pathological behavior [15, 19] without the need of extensive computational resources. However, hitherto, computational studies of ischemia have not included the inherent variability that is naturally observed in humans at the cellular electrophysiology level [20]. Including variability in mathematical models for ischemia could enable the creation of simulated databases that may be large enough for the training of machine learning algorithms.

To analyze the effects of inter-subject variability at the cellular level, recent research in cardiac EP has resorted to ‘experimentally-calibrated population of models’ (ePoM). The ePoM approach has been successfully applied to investigate the effects of variability in the EP properties of myocytes, both in physiology and pathology [21–25]. Previous population-based studies have explored how EP properties are affected by ionic current variabilities during acute ischemia [26, 27], but population-based techniques are yet to be adapted to create populations of ischemic ECG signals that include inter-subject variability.

This work presents a methodology that intends to prove the feasibility of ischemia classification using machine learning techniques. Here, simulated data, constructed using an ePoM approach, is used to validate proof-of-concept classifiers as a step prior to training with clinical data; the motivation behind this is that a classifier that does not work on ideal data will be uncapable of generalizing to a real-life environment. Morevover, this work hypothesized that training on simulated data could highlight which ECG biomarkers provide more information about the presence of ischemia. Consequently, an initial computational study could be used to guide the complicated analysis of real ECG signals. Observe that this does not mean that the discoveries from the computational study would be directly translatable to a clinical setup. As with any *in silico* study, the insights found through this framework would then require clinical validation.

## Materials and methods

### Models of human cellular electrophysiology

In this work, cardiac EP was simulated with the two most widely used, state of the art, models. First, the ten Tusscher-Panfilov 2006 (TP06) model [28, 29] provided an efficient implementation, capable of reproducing the effects of ischemia. For the purpose of comparison, the O’Hara-Rudy (ORd) model for the undiseased human cardiac ventricular action potential [30] was also used because it is the most detailed and thoroughly validated description of cardiac EP to date. However, since the ORd model is more complex and, consequently, computationally more expensive than the TP06, it was only used during the modeling part of this work and not for the machine learning section. The ORd model is not capable of reproducing conduction of action potentials (AP) under severe hyperkalemic conditions, so the equations for the *I*_*Na*_ *h* gates of the ORd model were modified as suggested by Passini et al. [31] and the time constants of the *h* and *j* gates were modified as prescribed by Dutta et al. [15]. Additionally, the sodium conductance was modified to *G*_*Na*_ = 47 mS/*μ*F to compensate for the changes in magnitude in *I*_*Na*_ that these new definitions produce.

### Experimentally-calibrated populations of models

Here, variability was investigated by means of an ‘experimentally-calibrated population of models’ (ePoM) approach. The population of models generation and calibration process has been explained in previous, successful, population-based studies in cardiac EP modeling [20, 24, 27]. One ePoM was created with each model using the experimental data presented in Table 1, obtained from the work by Morgan et al. [32]. These EP measurements were made on patients undergoing an electrophysiological study that revealed no evidence of heart disease; therefore, these populations were called *Control* ePoM.

**Table 1:** Monophasic action potential duration (MAPD) data used for calibration of the Control ePoM.

Drive cycle length	MAPD range
430 ms	170–240 ms
600 ms	195–290 ms

In short, each *Control* ePoM was generated as follows. First, as explained by Muszkiewicz at al. [20], all the maximal conductances and peak currents of the model in question were allowed to vary between 0 and 2 times their baseline value and 10000 different parameter combinations were selected using Latin Hypercube Sampling [33]. Then, using each parameter combination, a single cell simulation was performed at 430 ms and 600 ms cycle length (CL) to match the experimental calibration data. Each model was stimulated for 200 beats, needed to reach steady state conditions, and only the last beat was saved for further analysis. Once the 10000 simulations were completed, models were excluded from the study using the following criteria: action potential amplitude less than 0, resting membrane potential larger than -64 mV and upstroke time exceeding 10 ms. The exclusion criteria correspond to measurements that are outside of the range that is considered healthy. Finally, from the remaining models, only those that produced action potential duration at 90% repolarization (APD90) within the range specified in Table 1, for both CL, were kept; the resulting population of models gives the single cell *Control* ePoM.

To investigate variability in the conduction velocity and pECG biomarkers, each parameter set contained in the single cell *Control* ePoM was used to simulate AP propagation on a cable of cells. The population that resulted from these simulations was the cable *Control* ePoM. Each cable was formed by a homogeneous array of cells, its length was *L* = 2 cm, the inter-cell separation was Δ*l* = 0.02 cm and the cell-to-cell conductivity was *D* = 1.171 * 10^−3^ cm^2^/s. Stimulation was provided at one end of the cable (*l* = 0) at CL = 600 ms and for 30 beats to guarantee a steady state response; as before, only the last beat was saved. Pseudo-ECG (pECG) signals were calculated using the steady state response of the cables of cells using the equation provided by Gima and Rudy [19, 34]:

$$\begin{array}{c} \Phi_{e}\left(l^{\prime }\right)=A\int \left(-\nabla V\right)[\nabla \frac{1}{l-l^{\prime }}]dl \end{array}$$ (1)

where Φ_*e*_(*l*′) is the pECG observed on a virtual probe placed at position *l*′, *A* is a constant which depends on the radius of the fiber and the intra- and extra-cellular conductivities and *V* is the steady state AP on the cable of cells. Pseudo-ECGs were calculated on a virtual probe 2 cm away from the end of the cable (*l* = 4 cm) and only cells 15 to 85 were used to calculate the pECG; the latter prevents stimulation and boundary interference on the pECG signal [19]. Furthermore, *A* was set to 1 because the scaling of the pECG was not relevant for this study; in particular, the amplitude of the ECG is irrelevant in the design of predictive biomarkers because it depends on the size and constitution of the patient’s chest. As will be explained later, the pECG amplitude will be studied as it varies in time, factoring out the effects of the differences in chest morphology.

### Modeling variability in acute ischemia

The parameters contained in the *Control* ePoMs were then used to investigate variability in EP measurements under acute ischemia. Namely, each parameter set was used to solve a model that included the main effects of ischemia as suggested by previous work [15]. First, hyperkalemia was reproduced by increasing the extracellular potassium concentration ([*K*^+^]_*o*_). Second, acidosis was included in the model by inhibiting the fast-inward sodium (*I*_*Na*_) and L-type calcium (*I*_*CaL*_) currents through a constant (*f*_*inhib*_) that multiplied their computed value. Finally, hypoxia was modeled by including an additional ionic current that reproduces the effect of ATP-sensitive channels (*I*_*k*(*ATP*)_); the definition provided by Dutta et al. [15] was implemented:

$$\begin{array}{c} I_{k(ATP)}=f_{k(ATP)}*G_{k(ATP)}*(\frac{{\left[K^{+}\right]}_{o}}{{\left[K^{+}\right]}_{o_{n}}}{)}^{0.24}*\left(V-E_{k}\right) \end{array}$$ (2)

where the amplitude of the current depends on the ratio between the extracellular potassium concentration ([*K*^+^]_*o*_) and its control value (${\left[K^{+}\right]}_{o_{n}}=5.4$ mM), the transmembrane potential (*V*) and the potassium equilibrium potential (*E*_*k*_). The value of the channel conductance (*G*_*k*(*ATP*)_) was fixed at 0.064 mS/*μ*F and a scaling factor (*f*_*k*(*ATP*)_) was used to regulate the severity of the ischemic event.

To investigate the evolution in time of acute ischemic events, alongside variability, two ischemic severities were simulated using the parameters specified in Table 2. The *Severe* parameters were obtained from the previous work by Dutta et al. [15], and the *Mild* condition was assumed to be at a mid-point between the absence of ischemic effects and the severe ischemic parameters.

**Table 2:** Parameter sets for simulating the different stages of ischemia.

Parameter	Mild	Severe
*f*_*k*(*ATP*)_	0.1	0.2
*f*_*inhib*_	0.875	0.75
[*K*^+^]_*o*_	6.25 mM	9.0 mM

Single cell simulations were performed, with the same simulation parameters as before, to construct single cell *Mild* and *Severe* ePoMs. Then, several cable simulations were performed, allowing one to investigate the influence of the size of the ischemic region on EP biomarkers. In each cable of cells, an ischemic region was defined around the middle of the fiber, with a transition region of 0.1 cm in length at either end of the ischemic region. In the transition region the ischemic parameters were varied linearly from their ischemic value (*Mild* or *Severe*) to the *Control* value. Four sizes of the ischemic region were investigated (i.e. 0.5 cm, 1 cm, 1.5 cm and 2 cm), one cable *Mild* ePoM and *Severe* ePoM was produced for each. Each cable simulation was conducted using the same simulation parameters detailed before. Because of the long computational times, the ORd model was used only to perform the cable *Mild* (2 cm) and *Severe* (2 cm) ePoMs simulations.

### AP and pECG biomarkers

**Fig 1:**
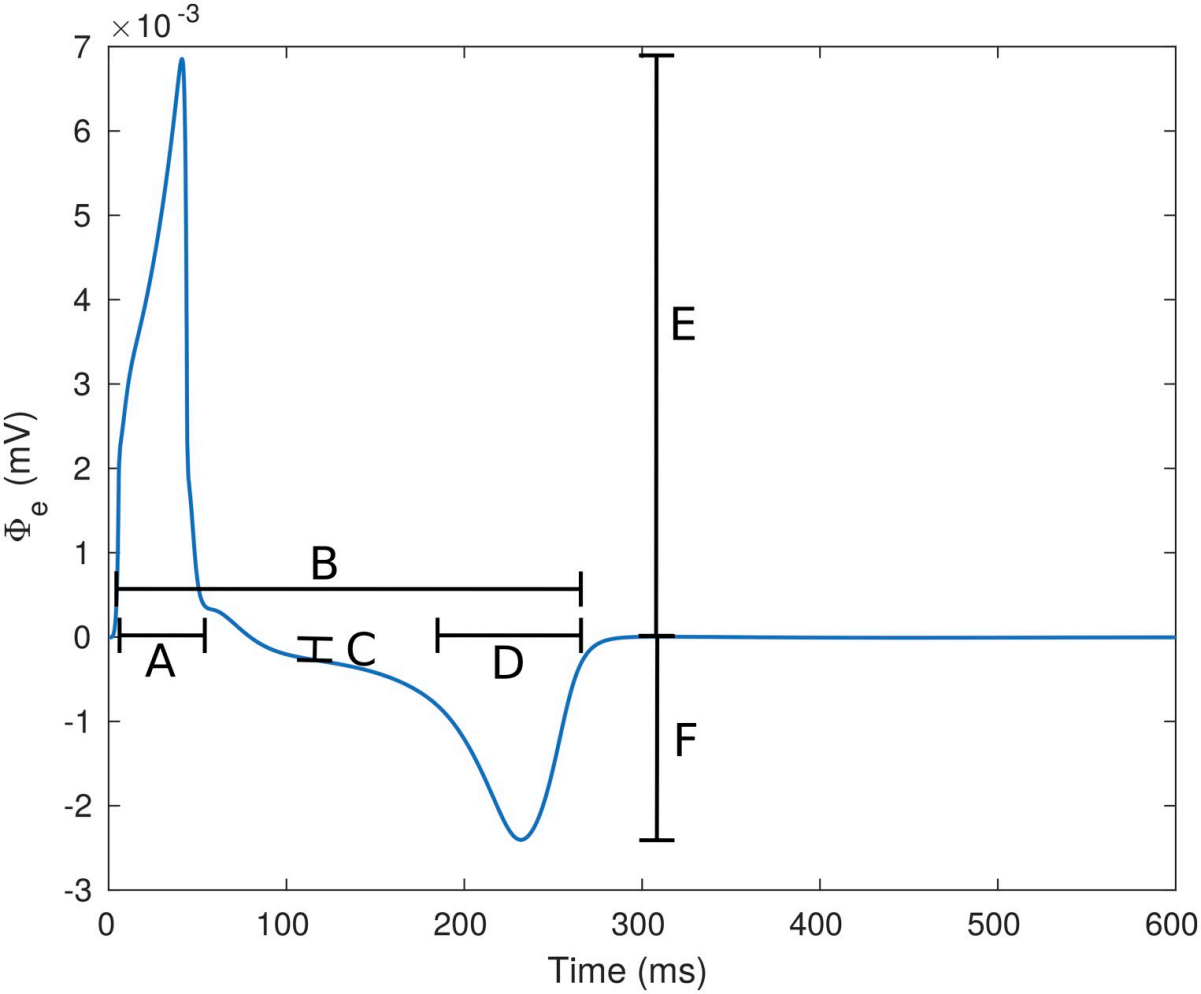
Pseudo ECG signal with its measured biomarkers. The pECG signal was calculated from a cable of TP06 cells. The biomarkers shown are: (A) QRS duration, (B) QT interval, (C) ST deviation, (D) T wave duration, (E) QRS amplitude and (F) T wave amplitude.

The effects of variability in EP properties under acute ischemic conditions were investigated using four action potential biomarkers and six pECG biomarkers. First, Action Potential Amplitude (APA) was measured as the amplitude of the transmembrane potential at the end of the initial depolarization phase; the amplitude was measured with respect to zero. Then, Action Potential Duration (APD90) was measured as the time when the transmembrane potential was at 10% of the APA. Also, the Resting Membrane Potential (RMP) was measured as the transmembrane potential at the end of the cycle. Additionally, only in the cable simulations, the Conduction Velocity (CV) was calculated as:

$$\begin{array}{c} CV=\frac{l_{2}-l_{1}}{AT\left(l_{2}\right)-AT\left(l_{1}\right)} \end{array}$$ (3)

where *l*_2_ = 1.6 cm, *l*_1_ = 0.4 cm and the Activation Time (*AT*(*l*)) at each of those points was obtained as the time instant when the transmembrane potential surpassed zero. Subsequently, from each cable simulation, the following pECG biomarkers were measured: (A) QRS duration as the time difference between the end and the beginning of the QRS complex, (B) QT interval as the time difference between the start of the QRS complex and the end of the T wave, (C) ST deviation as the amplitude of Φ_*e*_ at the mid-point between the end of the QRS complex and the beginning of the T wave, (D) T wave duration as the time difference between the start and the end of the T wave, (E) QRS amplitude was measured as the maximum value observed during the QRS complex, and (F) T wave amplitude as the maximum value observed in Φ_*e*_ during the T wave. An example of a pECG signal and the biomarkers can be observed in Fig 1. The onset and end of the waves were taken as the time instant when Φ_*e*_ reached 10% of their peak values at the relevant side of the wave.

Additionally, the ratio of change of each biomarker with respect to its control value, as ischemia developed, was calculated as:

$$\begin{array}{c} b^{\prime }=\frac{b_{I}}{b_{C}} \end{array}$$ (4)

where *b*′ was the ratio of change of a given biomarker, *b*_*C*_ was the biomarker calculated from the pECG of a control model and *b*_*I*_ was the biomarker calculated from the pECG of the same model after applying an ischemic variation (*Mild* or *Severe*). Observe that by calculating the ratio of change of the amplitude of the pECG, the effect of the electrical signal propagating through the chest (i.e. *K* in the pECG calculation) is factored out; hence, the study of the temporal evolution of the QRS and T wave amplitudes is relevant.

### Automatic classification of ischemic severity

Artificial Neural Networks (ANN) were trained to detect and classify the evolution of an ischemic event. The ANNs were fully-connected multi-layer perceptrons, with rectified linear hidden units and sigmoid output units. A complete exposition of the machine learning techniques used here has been made by Goodfellow et al. [35] and complete details of the implementations made for this work are provided in S1 File. Three datasets were used to train the models: the first group contained the pECG features measured from the cable *Control* ePoM of TP06 models, the second contained the pECG features from the cable *Mild* (2 cm) ePoM of TP06 models and the third group contained the pECG features from the cable *Severe* (2 cm) ePoM of TP06 models. Two different learning structures are proposed in this work.

The first approach (ANN_1_) was trained to discriminate between the three different populations of virtual signals (*Control, Mild, Severe*), which were encoded using one-hot vectors. This network was designed to use the magnitudes of the pECG biomarkers as inputs. Propagation through the network resulted in the activation (*o*_*i*_ = 1) of one of three output neurons (*o*_1_, *o*_2_ or *o*_3_) whilst the other two neurons remained inactive (*o*_*i*_ = 0). Then, classification was made by assigning each activation patterns (*o*_1_*o*_2_*o*_3_ = 100, *o*_1_*o*_2_*o*_3_ = 010, *o*_1_*o*_2_*o*_3_ = 001) to each of the populations (*Control, Mild, Severe*) respectively.

The second approach was a cascade of two networks. The first network (ANN_21_) would discriminate between a non-ischemic (*Control*) and an ischemic (*Mild* or *Severe*) signal using the magnitudes of the pECG biomarkers. The second network (ANN_22_) distinguished between the *Mild* and *Severe* ischemic signals using the ratio of change between the biomarkers. Each of these networks had a single output neuron.

All the networks were trained following the same procedure. First, the examples were randomly divided into a training set and an evaluation set, containing 75% and 25% of the data, respectively. The training set was then used to perform a 10-fold cross-validation. In each of the cross-validation iterations the weights of the ANN were initialized to random small values. Then, all the cross-validation training examples were propagated through the network and the cost was calculated using a binary cross-entropy function regularized using weight decay. The derivative of the cost with respect to each of the network’s weights was calculated using back-propagation and the weights were updated following the gradient descent. The weights that performed best during the cross-validation were tested in the evaluation set, the performance on this set was the one reported in the results. Complete details about the implementation of the neural networks and the training algorithm are provided as Supporting Information (S1 File).

Each ANN was repeatedly trained, varying the number of hidden layers and hidden neurons per layer, until the best performing network (in the evaluation set) was found. The performance of each trained model was measured using the Positive Predictive Value (PPV) and Sensitivity (Se) and the ANNs were compared using the F_1_-score. The hyperparameter search method implemented here is largely similar to those that have been used to achieve state-of-the-art performances in ECG classification [36–39]; to date, this is the best known methodology to find generalizable hyperparameters. Finally, after finding the optimal topology for each network, the relative importance of the input features in each classification task was found using the approach explained by Garson [40] and Goh [41].

To provide a baseline value for the classification performance, a logistic regression classifier was also trained. This was done in order to establish that the classification on the pECG signals was not trivial and to clearly illustrate that complex non-linear relationships exist in the data. The results from the logistic regression are part of the Supporting Information.

### Implementation details

The solutions to the TP06 and ORd models were implemented in Matlab (R2017a) and solved using ode15s. The Matlab solver uses a variable order method to solve stiff ordinary differential equations, it was configured to use a maximum relative tolerance of 10^-3^, a maximum absolute tolerance of 10^-6^ and the default maximum step size (i.e. 0.1|*CL*|). This last option is recommended by Matlab because reducing it can significantly slow down the routine; moreover, the results had sufficient quality without limiting the maximum step size of the solver. The implementation of both models was based on the codes provided by the authors of the original papers (TP06: http://www-binf.bio.uu.nl/khwjtuss/SourceCodes/, ORd: http://rudylab.wustl.edu/research/cell/code/AllCodes.html). The implementations were adapted to simulate populations of models and ischemia as explained in the previous sections. The reaction-diffusion system used for the cable simulations was solved using the numerical method of lines [42]. Namely, the spatial derivative was discretized using a central difference approximation that converts the PDE system into an ODE system to be solved using ode15s. These routines were deployed in the UCL Computer Science cluster (hpc.cs.ucl.ac.uk) to compute all the models needed to build the previously described populations of models.

The artificial neural network routines were implemented in Python (3.6.1), using NumPy (1.13.1) and Theano (0.9.0). The training and evaluation was made in a DELL Precision Tower 5810 with an Intel Xeon E5-1620 CPU, 32GB of RAM, and an Ubuntu (16.04 LTS) operating system.

## Results

### AP and pECG biomarkers of *Control* populations

The calibration process resulted in a single cell *Control* population of 2044 TP06 models and 2226 ORd models. The values of the biomarkers measured from the single cell *Control* AP traces, at 600 ms cycle length, and from the pECGs calculated from the cable *Control* ePoMs are presented in Table 3. The pECG biomarkers measured as magnitudes are not presented because, as explained in the methods, these markers were only interesting to observe as ratio of change with respect to the control value.

**Table 3:** AP and pECG biomarkers measured from the Control ePoM. The values are presented as mean and standard deviation.

Biomarker	TP06	ORd
APD90 (ms)	233.28 ± 34.87	227.73 ± 19.83
APA (mV)	52.47 ± 16.35	37.90 ± 7.08
RMP (mV)	−84.91 ± 2.33	−87.70 ± 0.92
CV (cm/s)	60.80 ± 15.17	50.14 ± 6.34
QT interval (ms)	264.30 ± 26.84	273.03 ± 26.19
QRS duration (ms)	22.12 ± 14.50	29.04 ± 10.05
T wave duration (ms)	33.78 ± 12.21	50.56 ± 28.76

### AP biomarkers of ischemic populations

**Fig 2:**
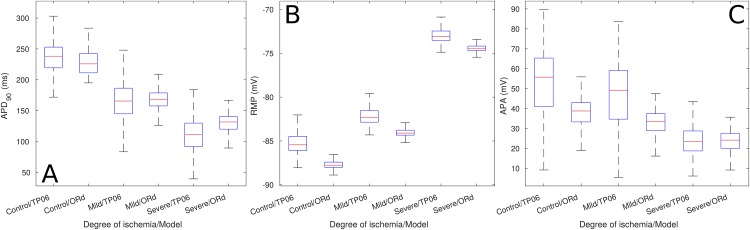
Biomarkers of single cell ePoMs. The image shows the biomarkers measured at 600 ms cycle length. (A) Action potential duration at 90% repolarization, (B) resting membrane potential and (C) action potential amplitude.

**Fig 3:**
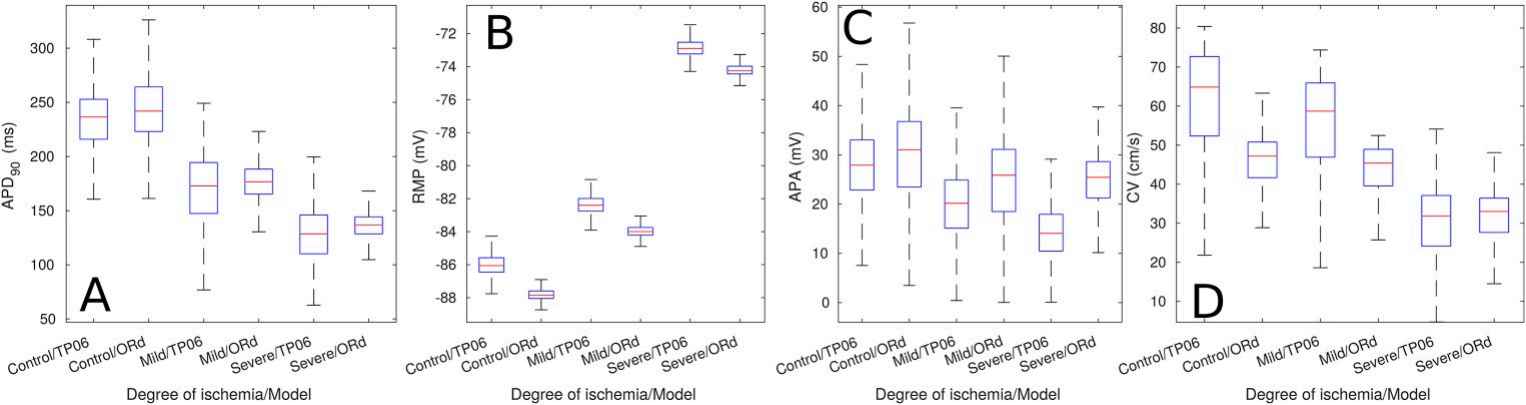
Biomarkers of cable ePoMs, size of the ischemic region: 2 cm. The boxplots include the biomarkers measured on all the cells of the cable. (A) Action potential duration at 90% repolarization, (B) resting membrane potential, (C) action potential amplitude and (D) Conduction velocity.

Fig 2 shows the magnitude of the AP biomarkers of the single cell *Mild* and *Severe* ePoMs. Save for a small difference in magnitude, both models showed similar results: the single cell *Mild* ePoMs were characterized by a decrease in APD90, an increase in RMP and a decrease in APA. These variations were accentuated when the biomarkers were measured in the single cell *Severe* ePoM. The trends observed in the AP biomarkers of the single cell simulations were also observed in the AP biomarkers measured from the cables of cells (see Fig 3). Additionally, there was a minor reduction in conduction velocity in the cable *Mild* (2 cm) ePoMs, but the reduction became evident when the CV was measured in the cable *Severe* (2 cm) ePoMs.

### pECG biomarkers of ischemic populations

A qualitative assessment of the pECG signals a clear reduction in the QT interval, an increase in the length of the T wave and ST segment deviation from the isoelectric line, all of which are well-known markers for ischemia in ECG recordings. Additionally, an increase in the QRS complex duration under *Severe* ischemic conditions for all signals was observed. However, this last observation was only evident in some of the signals that simulated *Mild* ischemia; this is a clear manifestation of inter-subject variability. Examples of the pECG traces obtained in this work are provided as Supporting Information (S2 File).

**Fig 4:**
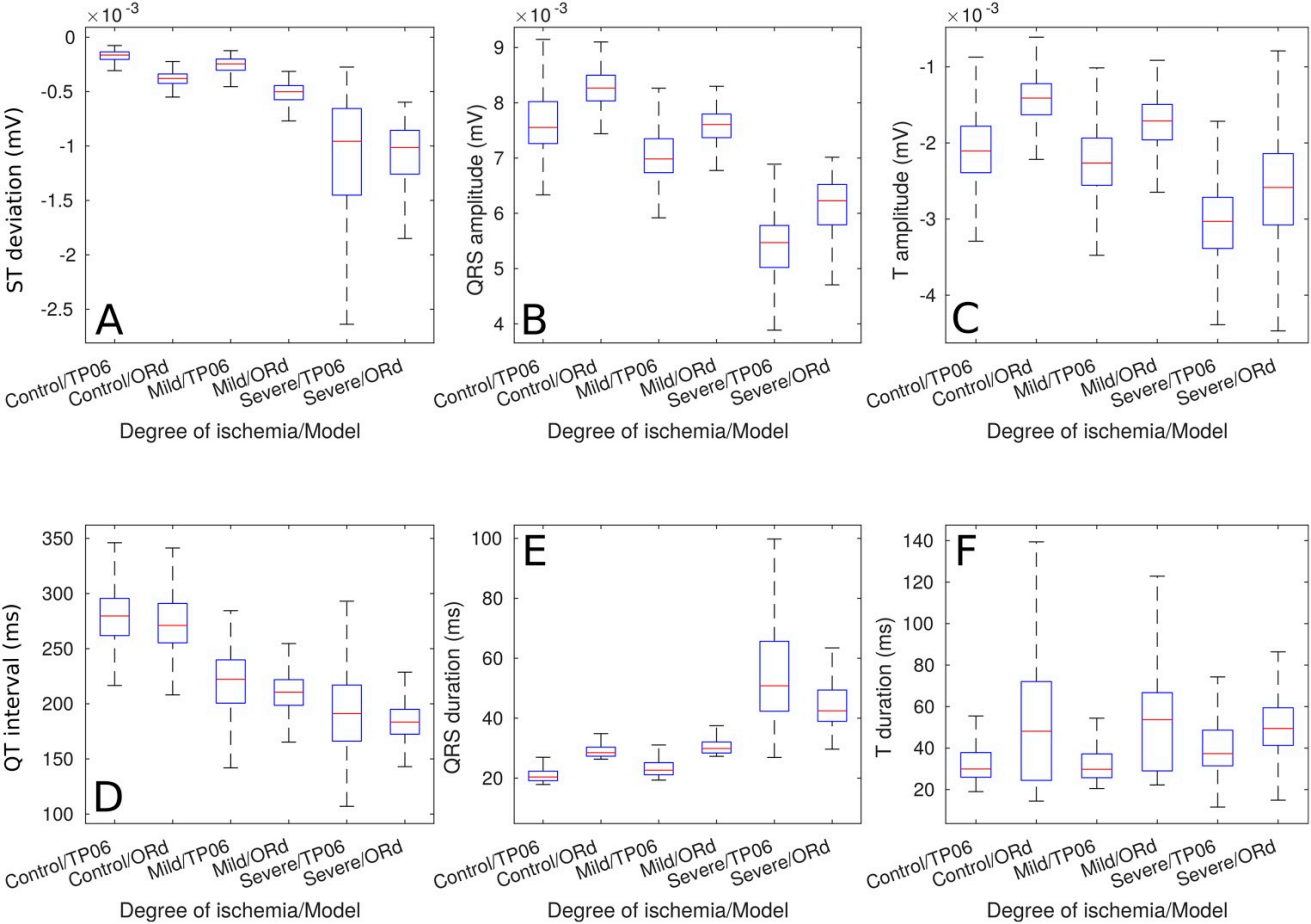
Pseudo-ECG biomarkers of cables of ePoMs, ischemic region: 2 cm. (A) ST deviation, (B) QRS amplitude, (C) T wave amplitude, (D) QT interval, (E) QRS complex duration and (F) T wave duration.

**Fig 5:**
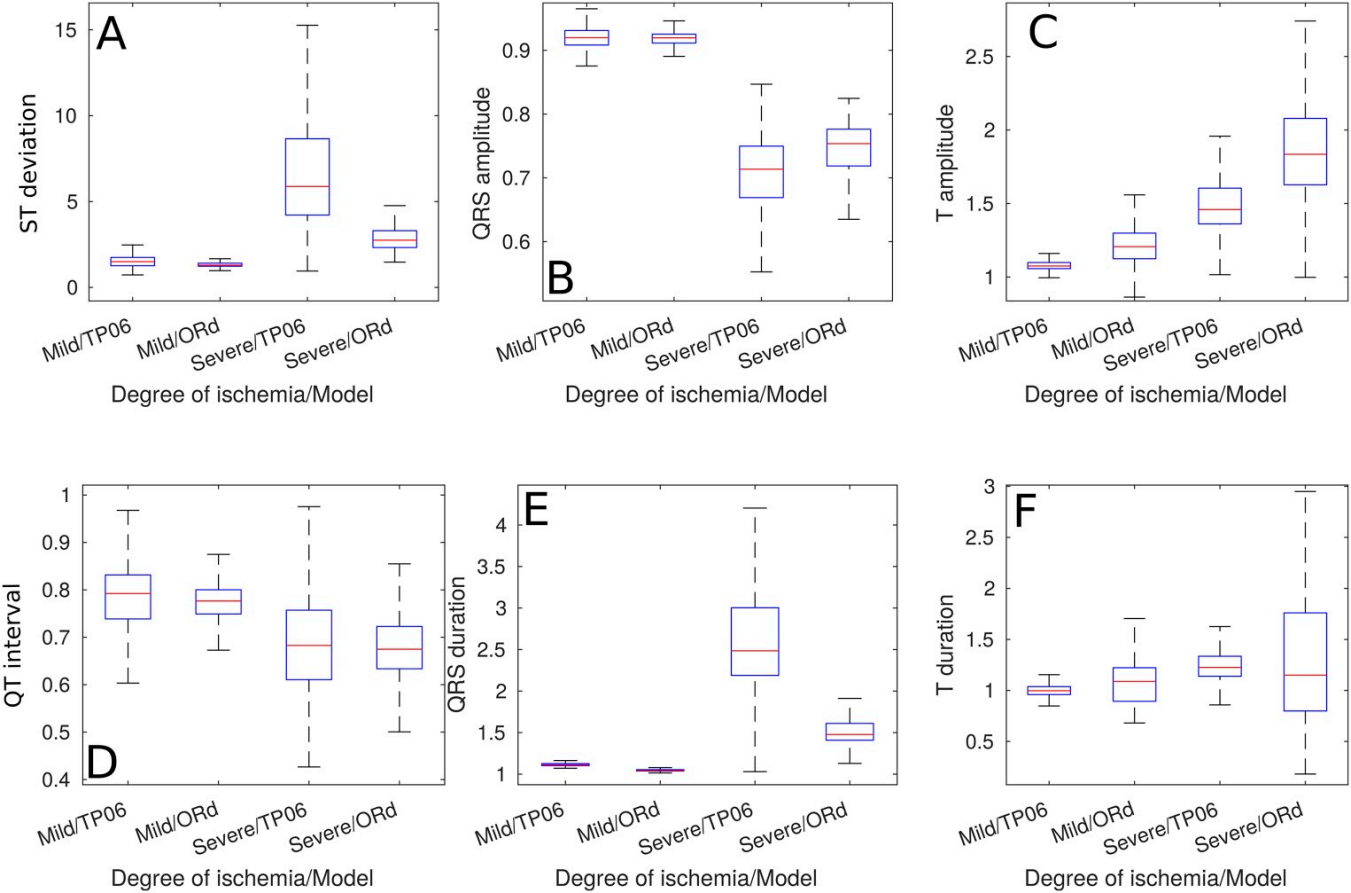
Ratio of change of pseudo-ECG biomarkers of cable ePoMs, ischemic region: 2 cm. (A) ST deviation, (B) QRS amplitude, (C) T wave amplitude, (D) QT interval, (E) QRS complex duration and (F) T wave duration.

The aforementioned trends can be quantitatively observed in Fig 4, these biomarkers were measured from the 2 cm ischemic cables of cells. The only biomarker that showed a clear difference between the *Control* and the *Mild* cases was the QT duration, all the other biomarkers showed evident deviations from the *Control* biomarkers only in the *Severe* ischemic case. The main trends that underpinned the presence of a *Severe* ischemic event were an increase in T-wave magnitude, pronounced ST deviation, reduced QRS amplitude, reduced QT duration, increased QRS duration and an increase in T wave duration—they were equivalent in the results from both models. Furthermore, Fig 5 shows that the previously mentioned trends in the magnitudes of the biomarkers were more evident when analyzing their ratio of change.

**Fig 6:**
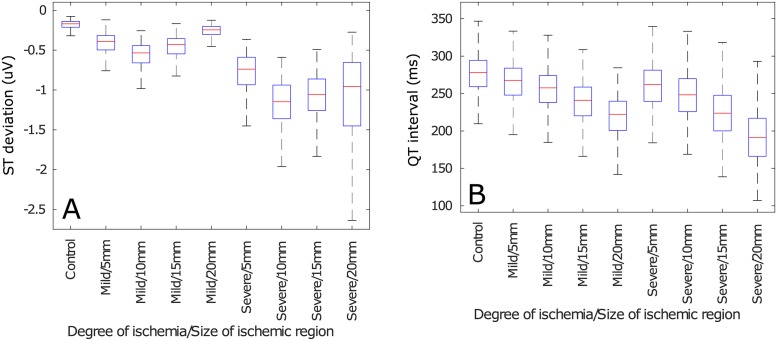
Pseudo-ECG biomarkers of TP06 cables of cells as a function of the size of the ischemic region. (A) ST deviation, (B) QT interval.

**Fig 7:**
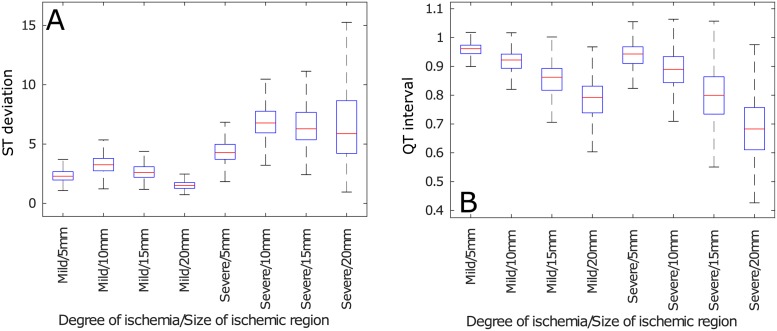
Ratio of change of pseudo-ECG biomarkers of TP06 cables of cells as a function of the size of the ischemic region. (A) ST deviation, (B) QT interval.

The influence of varying the size of the ischemic region upon the the pECG biomarkers can be observed, only for two representative features, in Figs 6 and 7 for the magnitudes and ratio of change, respectively. These figures show two different trends as a function of the size of the ischemic region. First, the biomarkers that depend on depolarization (i.e. QRS amplitude, QT interval and QRS duration) increased, or decreased, linearly with respect to the size of the ischemic region. Second, the magnitudes, or ratio of change, of the biomarkers measured after the depolarization ends (i.e. ST deviation, T wave amplitude and T wave duration) had a parabolic shape with respect to the size of the ischemic region. The results for all the features can be consulted in the Supporting Information (S2 File).

### Neural networks classification performance

The database of TP06 *Control*, *Mild* (2 cm) and *Severe* (2 cm) models, which included variability, created by means of populations of models enabled the training of the ANNs that would detect and classify ischemic events. The results of training the ANNs are presented in Table 4, where HL stands for number of hidden layers and HU for number of hidden units per layer. The cascade of neural networks (ANN_21_ followed by ANN_22_) showed higher performance than the single multi-class classification network (ANN_1_). Notwithstanding, ANN_1_ achieved high performance with significantly less complexity than the combination of ANN_21_ and ANN_22_. Also, the learning rate required to train ANN_1_ was 20 times smaller than the one required to train ANN_22_ and half of that required for ANN_21_. The significant reduction in learning rate meant that training ANN_1_ involved considerably longer times than training the other two.

**Table 4:** Best performing neural networks.

Network	HL	HU	Se (%)	PPV (%)	F_1_-score (%)
ANN_1_	5	7	94.77	95.52	95.15
ANN_21_	5	8	99.00	96.28	97.62
ANN_22_	4	4	100	100	100

### Relative importance of pECG features

The relative importance of each feature, for each of the optimal networks in Table 4, is presented in Table 5. The table shows that the most relevant features for the multi-class classification network were the QT interval and the QRS duration, the T wave amplitude was the least relevant and the other features shared similar importance. In the classification between *Control* and ischemia it was the QT interval that was most important, the ST deviation the least important and all other features shared equal relevance. Finally, when discerning between *Mild* and *Severe* ischemia, it was the relative variation in amplitude of the QRS and T waves which proved to be more relevant and the duration of the T wave the least relevant feature.

**Table 5:** Relative importance of the input features in each ANN.

Network	ANN_1_	ANN_21_	ANN_22_
QT interval	0.24	0.28	0.15
ST deviation	0.16	0.09	0.12
QRS amplitude	0.14	0.17	0.29
T amplitude	0.09	0.16	0.21
QRS duration	0.21	0.18	0.13
T duration	0.15	0.12	0.09

## Discussion

First, the work presented in this paper investigated the role that inter-subject variability, at the cellular level, plays when detecting ischemia through ECG signals; this was done through a computational approach, using pseudo-ECGs. Two virtual databases, containing over 6000 models, were constructed by means of an experimentally-calibrated populations of models approach. The AP and pECG biomarkers measured from the models contained in the database were in concordance with various simulation and experimental data [19, 43–46]. To the best of our knowledge, this is the most comprehensive simulation database for the study of acute ischemia to date, it includes cellular-level variability and contains information about the presence of ischemia, its severity and its effect on the ECG. Second, this study has introduced the use of simulation data to validate proof-of-concept neural networks that could, with further research, inform subsequent clinical studies in the early detection of ischemic events using ECG biomarkers; this approach had been, to date, unexplored. The analysis of the neural networks highlighted the importance of using multiple biomarkers and complex features for the detection of ischemic events and shed light on which are those that should be monitored for early detection. The mathematical models and machine learning techniques developed throughout this study will enable further research in the detection and prevention of myocardial ischemia.

### ePoMs reproduce physiological variability under control and ischemic conditions

Two virtual databases were constructed using experimentally-calibrated populations of models, one using TP06 models and the other using ORd models. The results presented in the previous section indicate that the models contained in these databases reproduce the healthy behavior of cardiac myocytes and the evolution of an ischemic event within those individual models. Furthermore, the ePoM approach enabled the introduction of inter-subject variability in those virtual databases, thus making them closer to what would be found in a clinical study. The results also show that variability has an additive effect, i.e. a relatively small variability in AP biomarkers resulted in larger variability in pECG biomarkers.

Table 3 shows that the AP biomarkers of the *Control* ePoMs were within the values expected in healthy cells. Additionally, the pECG biomarkers were consistent with those previously presented by Gima and Rudy [19]. Hence, it can be argued that both of the *Control* ePoMs were, indeed, a good representation of a healthy population. Furthermore, even though the populations were constrained to a specific range of APD90, the *Control* biomarkers (see Table 3) vary within individual models; this is the effect of including inter-subject variability.

Fig 2 exemplifies how AP biomarkers were affected during ischemic events whilst still showing inter-subject variability. Namely, the impairment in *I*_*CaL*_ and introduction of hypoxic effects (through *I*_*k*(*ATP*)_) produced a clear reduction in action potential duration, the increase in extra-cellular potassium translated in an increase in RMP and the reduction of the *I*_*Na*_ current caused a clear reduction in APA. Furthermore, Fig 4 shows that the variations from the *Control* values observed in the ischemic pECG biomarkers were similar to those described in clinical practice. Namely, the *Mild* and *Severe* ePoMs showed ST segment deviation [43, 45], reduction in QT interval [43, 44] and T wave deformation [45–47]. The variability observed at the cellular level during the ischemic events was relatively small, Fig 2 shows little overlap in the values of APD90 and RMP. However, the additive effect of variability can be clearly observed in Figs 3 and 4, where there is a larger overlap in the values of conduction velocity and pECG biomarkers.

These results are consistent with previous pECG studies [14, 16, 17, 19] and with well-known effects of ischemia on humans [43–46]. So it can be argued that the variability contained in the ePoMs is a faithful representation of the actual variability observed in humans. One of the key contributions of this work is providing two virtual databases that model the evolution of ischemic events, where each model represents *different* physiological conditions. These databases could be used in future studies to find relationships between ionic current variabilities, ischemia and pECG biomarkers.

### Variability precludes the classification of ischemia through a single biomarker

Our results suggest that variability makes it impossible to determine both the presence and severity of ischemia through a simple analysis of the values observed in the pECG biomarkers, which was the first hypothesis of this work. This is a consequence of the additive effect of variability, the ECG signal is produced from the aggregation of the electrical responses of many individual cells, so the relatively small variability observed in cellular biomarkers (i.e. APD90, APA and RMP) is magnified when measuring the ECG biomarkers.

In Fig 2, one can observe that the 1st and 3rd quartiles of the distributions (i.e. the upper and lower limits of the boxplots boxes) of APD90 and RMP showed no overlap. Hence, the different ischemic severities could be classified, at the cellular level, by means of thresholds; but these measurements are not routinely made in patients. Fig 4 shows that all the measured pECG biomarkers presented overlap in the 1st and 3rd quartiles when comparing *Control* to *Mild* or *Mild* to *Severe*. So, it is concluded that the additive effect of variabilities makes it difficult to draw the lines that classify between the different populations. For example, QRS complex prolongation was observed in some *Mild* ischemic pECG signals, but the same effect was only observed in *Severe* cases of other models. This early QRS complex prolongation was observed in models that had a weak *I*_*Na*_ and/or *I*_*CaL*_ in their *Control* values. These can be regarded as examples of particularly vulnerable patients in which a clinician might have mistakenly diagnosed a Severe ischemic event when, in reality, it was Mild. If, alternatively, the *Mild* models were used as the classification gold-standard, all other patients would be diagnosed late.

An additional source for variability, that confuses the classification of ischemic events, can be observed in Figs 6 and 7. The biomarkers that measured the QRS complex behaved linearly whilst the ones that measured the T wave behaved parabolically with respect to the size of the ischemic region. These behaviors emerged because the QRS complex mainly depends on the velocity of the depolarizing wave, and the larger the ischemic region, the greater is the fiber’s depolarization time. Alternatively, the differences in T wave morphology are produced by the dyssynchrony in the repolarization of the cells along the fiber, so the fibers where repolarization was more homogeneous (i.e. those where the ischemic and non-ischemic regions had similar size) had similar T wave markers.

One can observe, from Fig 5, that inter-subject variability did not preclude a threshold-based classification when analyzing the ratio of change in the biomarkers; this is because the ratio of change is a more complex feature than the magnitude and, consequently, carries more information. Nevertheless, this did not resolve the problem of determining if the pECG that was being observed corresponded to a healthy heart or to a diseased one. Also, using the ratio of change makes it necessary to have control values for a patient so one can determine if the biomarker has changed and, intuitively, determining a baseline value for a patient is not at all trivial. Furthermore, the variability introduced by considering a variable size for the ischemic region still made it impossible to define clear thresholds or rules, even when assessing the the ratio of change in the biomarkers (see Figs 6 and 7).

It is important to highlight that in the simplified case of a homogeneous cable of cells, the features measured in the pECG are a direct consequence of those measured in the individual cells. For example, the QRS duration is simply an inverse measure of the conduction velocity and the QRS amplitude is a result of combining the APA and the CV. However, once the ischemic formulation is included, this analysis does not hold anymore. This is particularly evident from the different behaviors observed in Fig 6, where the size and severity of the ischemic region introduce non-linear relationships in the behavior of the pECG. Consequently, classification of ischemia becomes no longer trivial. Another key contribution of this paper is to present, through the use of pseudo-ECGs, that the variability originated at the cellular level is amplified when measuring ECG biomarkers, so simple threshold rules and visual inspection of signals will, invariably, result in poor detection and classification performance.

### Machine learning is effective in detecting and classifying ischemic events

On the one hand, the logistic regression classifiers, used as baseline, had poor performance in classifying *Control* or *Mild* models, they were only good when a *Severe* model was presented (please refer to S1 File for details), demonstrating that more advanced classifications algorithms are required. On the other hand, the neural network models produced high performance in all the classification tasks. It can be observed from the two classification paradigms that it was possible to use the pseudo-ECG features to classify the evolution of an ischemic event in a population of fibres. The first proposed approach (multi-class classification) proved to have good performance in assessing the severity of the ischemic event. The second classification topology presented improved performance over the first one, this can be regarded as a consequence of it using more complex features when distinguishing between *Mild* and *Severe* models. Furthermore, the latter topology had added robustness in the detection: if the first network produced a false positive, the second one could filter it by observing no change in the magnitude of the biomarkers, and false negatives were counteracted by the improved sensitivity.

The main advantage in learning the multi-class classification model is that it does not require patient history and it uses ECG biomarkers that can be easily acquired in a routine visit to the specialist consultant. However, incorrect classification was observed when the network was applied to models that are severe outliers; this was translated in relatively low sensitivity when compared to the second architecture. In the second scheme, the severity of ischemia was assessed by taking into account the changes in biomarkers within the model, hence, it can be regarded as a network that monitors the patient’s condition over time. The drawback of the cascade of ANNs is that it requires a history for the patient so that a control set of values can be established; moreover, it is not trivial to establish what this control value should be for a given patient and it is bound to change over time depending on parameters that may be too complex to model (e.g. lifestyle, ageing or co-morbidities).

### Relevance of pECG features in the detection and classification of ischemic events

Our results suggest that the use of several biomarkers is convenient when detecting and classifying ischemic episodes, as has been highlighted by previous research [48]. This is mainly because the biomarkers that were relevant for detection were not the same as those relevant for classification.

Clinical studies have previously shown that QT shortening is observed in the first 12 hours after the onset of acute myocardial infarction [43, 49] and Table 5 shows that the classification between *Control* and ischemia is mainly driven by the QT interval. Consequently, the results indicate that QT shortening should be the main cause for alarm in applications that monitor acute myocardial ischemia. However, this feature became less relevant when discerning between the *Mild* and *Severe* ischemic signals. The results indicate that the relative variation in the amplitude of the QRS complex and T waves are the features that will play an important role in determining the severity of the ischemic event. Additionally, in the three networks there were at least three features of equal importance which, combined, were responsible for at least 40% of the classification. This means that even if a ‘heavy’ feature is absent or mistaken the classification can be made using the other features.

Interestingly, the gold-standard for ischemia detection (i.e. ST segment deviation) may not be well suited for detecting ischemia at an early stage. Fig 4 indicates that its value under *Mild* ischemic conditions was similar to that observed in *Control* models and Table 5 confirms that the ST deviation is the least relevant feature when classifying between the *Control* and the ischemic cases (ANN_21_). Consequently, our results imply that, using the network architectures presented here, ST deviation only becomes relevant in the later stages of acute ischemia, when discerning between the *Mild* and *Severe* ischemic cases.

### A framework for testing proof-of-concept ischemia-detection methods

An additional contribution of this work was to prove that non linear functions constructed using machine learning techniques are capable of producing high classification and detection performance in an environment plagued by inter-subject variability. This has validated the use of the presented neural network architectures as a proof-of-concept for the early detection of ischemic events. Given the results, we can hypothesize that classification using machine learning models would be beneficial to the diagnosis and classification of acute myocardial ischemia. Indeed, the results shown in Tables 4 and 5 indicate that the AI models are capable of learning which pECG features are more relevant for the classification of the signal and, consequently, are capable of performing the task with higher accuracy.

The results presented here are still far from being applied in clinical practice. However, the results presented in this publication could be used as a guide for future works that may bring this approach closer to the clinic. As shown by the results, determining a patient-specific baseline value for ECG biomarkers should be the matter of further research because it could dramatically improve the detection of early-stage ischemia; indeed, the use of these, more complex, features enabled a significant increase in performance when compared to the multi-class classifier. Also, our results indicate that QT interval and QRS duration are more relevant for the detection of acute ischemic events; then, further studies should focus on further exploting these results and, subsequently, on making robust detection methods for these features because they are most-likely to have an impact on the AI models’ decision process. Finally, this study supports the use of computational, population-based, methods for testing proof-of-concept classification models when there are few data available and the introduction of machine learning techniques to aid clinicians when diagnostic methods are highly affected by inter-subject variability.

### Limitations

It is important to acknowledge that a 1D cable model entails a number of limitations because one is neglecting the effects of the 3D geometry, particular Purkinje activation sequences, the effects of chest anatomy, and so on. However, simplifying the pECG calculation to a 1D cable is necessary this case to make the population study tractable (i.e. simulating thousands of different ischemic conditions is currently impossible to perform, tractably, in a full-heart geometry). Also, the simplification of a homogeneous cable for the “healthy” case is necessary because of the nature of the mathematical models used. Both the TP and ORd models distinguish the epi, endo and M cells only by variations of their parameters. Since the ePoM explores variability through a variation of the parameters of the models, making a heterogeneous cable of cells may produce undesired confounding factors (e.g. ending up with epicardial cells at the beginning of the cable or with endocardial cells at the end). Given that the purpose of this study is to validate the use of computational models as a means to train machine learning algorithms, the aforementioned limitations are justifiable in this context.

Previous works have shown that the pseudo-ECG is capable of simulating real-life physiology and pathology [14, 16, 19], however, there are still some limitations concerning the clinical translation of the results presented in this work. First, the use of fibres of cells instead of whole-heart models means that the results obtained in this paper do not account for certain phenomena that can be observed in an ischemic heart; e.g. re-entrant circuits that lead to arrhythmia, changes in the direction of electrical propagation because of conduction block in the ischemic zone, patient-specific Purkinje activation sequences, 3D geometry-related variability and variations in the ECG depending on the position of the affected area and the characteristics of the patient’s torso. Second, the computational study gives the advantage of a noise-free environment that makes it simple to measure the pECG features; in a real-life acquisition, the quality of the acquired signal, and of the denoising techniques, will play an important role in measuring the biomarkers and is expected to have an impact in the performance of the machine learning models.

Also, an additional limitation is that, since only the last beat of each model is used, temporal variations in the ECG (e.g. due to cell-to-cell uncoupling) are not accounted for when training the machine learning methods; these could play an important role in the generalization of the models. Moreover, the results revealed that the relative variation in amplitude of the QRS complex and T wave are important to determine the severity of the ischemic event, but in patients this variation may be due to a change in thoracic morphology (e.g. because of weight loss/gain) instead of a pathological variation. Additionally, the only consequence of CHD that we can model nowadays is ischemia, which is not always present in CHD; further research is required if we hope to use approaches like the one shown here to detect every possible CHD case.

Finally, only Deep Learning algorithms were used for classification; although these are known to be universal approximators [50] and optimal classifiers [35], their interpretability is limited. Other ML approaches that may be more interpretable (e.g. Bayesian methods, support vector machines, decision trees) could be tested and their performance compared. These limitations will be addressed in future works, where real-life ECG databases will be used in concert with the simulated data presented here to validate these results and to create robust systems for the detection and assessment of severity of ischemic events.

## Conclusion

This paper has presented the creation of two virtual databases that contain models which capture the evolution of ischemic events in cardiac cells and include inter-subject variability. The databases were constructed using ‘experimentally-calibrated populations of models’. Furthermore, these virtual databases were used to train machine learning algorithms capable of detecting and classifying ischemic events with high performance. The entries of the databases include single cell *Control*, *Mild* ischemic and *Severe* ischemic models and cable *Control*, *Mild* ischemic and *Severe* ischemic models with varying sizes of the ischemic region. The study presented in this paper represents progress in the use of experimentally-calibrated populations of models as a means to investigate coronary heart disease. Four key contributions can be highlighted from this work. (1) The study on variability presented here would have been impossible to perform clinically; finding healthy individuals exemplifying all the physiological conditions contained in the *Control* ePoMs is unfeasible and inducing ischemia in these patients is unethical. (2) This paper has shown that the additive effect of variability precludes the detection and classification of ischemic events using simple thresholds or visual inspection; non-linear classifiers, capable of automatically assessing ECG signals, must be introduced as a means to help clinicians when diagnosing coronary heart disease. (3) Our results have shown that machine learning techniques could be a valid tool to solve the aforementioned challenge and have validated two different proof-of-concept neural network architectures that could be used in a future clinical study. (4) The analysis of the different neural network topologies revealed that the biomarkers that better detect ischemia and those that better assess its severity are different, so a multi-biomarker analysis is essential; in particular, future clinical studies should focus on how to determine a *Control* value for the biomarkers because calculating their ratio of change can significantly improve ECG classification. The synthetic databases and machine learning methods presented here form an important precedent because they can be used as a step prior to a clinical study where significant challenges may arise and identifying the sources for errors may be more complicated. The use of virtual databases to explore the effects of ischemia on cardiac cells and to validate proof-of-concept models for the early detection of coronary heart disease could give valuable information prior to a clinical study, thus saving time and speeding-up the applicaton’s development process.

## Supporting information

S1 File Training algorithm. Contains the details of the training algorithm and the performance of all the tested network topologies.
(PDF)

S2 File Supplementary figures. Contains the figures that complement the presented results and further support the discussion.
(PDF)
