# Supplementary material for: A modeling and machine learning approach to ECG feature engineering for the detection of ischemia using pseudo-ECG
Source: PLoS One. 2019 Aug 12;14(8):e0220294. doi: 10.1371/journal.pone.0220294 (PMC6690680; doi:10.1371/journal.pone.0220294)
Supplement: S1 File — Contains the details of the training algorithm and the performance of all the tested network topologies. (PDF) [file pone.0220294.s001.pdf]

Details of the training algorithm

Training data

The pECG feature data used to train the neural networks was formed by three datasets. The first was the TP06 cable *Control* ePoM, the second dataset was the TP06 cable *Mild* ePoM and the third set of signals was the TP06 cable *Severe* ePoM. The signals used in this work were the pECG calculated from the last beat of each model once a steady state response was achieved, as explained in the manuscript. Each of the three datasets contains 2044 signals, which means that a database of 6132 pECG beats was used for training. It should be observed that, given that this is a computational study, we are able to maintain the balance between the classes, this is favorable during training because it avoids biasing the network towards a given class and it improves the generalization of the model. Then, using the features measured from a given pECG signal, the learning task for each of the networks was to tell to which dataset the signal belonged to. For training, we define a design matrix $\mathbb{X}$ in which each row corresponds to an example and each column to a feature. Also, we define a target matrix $\mathbb{Y}$ in which each row correspods to the target for each of the training examples and each column to an element in the class-encoding vector; in this work we used one-hot encoding to produce the classification targets. The training steps are detailed in the following sections.

Network structure

In this work, the networks were implemented using a general framework that allows to train dense neural networks for any application. This framework is implemented to take advantage of the improved computation capabilities available when using matrix operations. Consequently, each epoch of the training process is done "in one go", so all the training examples are processed simultaneously. Additionally, a bias term is included in every layer, as is common practice in deep learning approaches. Consequently, the input to the network is a matrix of the form:

$$\mathbb{X}_b = \begin{pmatrix} 1 & x_{11} & x_{12} & \dots & x_{1M} \\ 1 & x_{21} & x_{22} & \dots & x_{2M} \\ \vdots & \vdots & \vdots & & \vdots \\ 1 & x_{N1} & x_{N2} & \dots & x_{NM} \end{pmatrix} \tag{1}$$

The input matrix ($\mathbb{X}_b$) has size $N \times (M+1)$, where N is the number of training examples, M is the number of input features and term x_{ij} corresponds to feature j of example i.

Then, the network performs an affine transformation of the inputs to project them into the first hidden layer. This requires a matrix of weights:

$$\Theta^{(1)} = \begin{pmatrix} \theta^{(1)}_{01} & \theta^{(1)}_{02} & \dots & \theta^{(1)}_{0S} \\ \theta^{(1)}_{11} & \theta^{(1)}_{12} & \dots & \theta^{(1)}_{1S} \\ \theta^{(1)}_{21} & \theta^{(1)}_{22} & \dots & \theta^{(1)}_{2S} \\ \vdots & \vdots & & \vdots \\ \theta^{(1)}_{M1} & \theta^{(1)}_{M2} & \dots & \theta^{(1)}_{MS} \end{pmatrix} \tag{2}$$

which has size $(M+1) \times S$, where S is the number of hidden neurons in the first hidden layer and term $\theta^{(1)}_{ij}$ will multiply input feature i when performing the affine transformation from the input layer into neuron j. Then, the affine transformation for all the training examples is done with the matrix multiplication:

$$\mathbb{Z}^{(1)} = (\Theta^{(1)})^T \cdot \mathbb{X}^T = \begin{pmatrix} z^{(1)}_{11} & z^{(1)}_{12} & \dots & z^{(1)}_{1N} \\ z^{(1)}_{21} & z^{(1)}_{22} & \dots & z^{(1)}_{2N} \\ \vdots & \vdots & & \vdots \\ z^{(1)}_{S1} & z^{(1)}_{S2} & \dots & z^{(1)}_{SN} \end{pmatrix}, \tag{3}$$

where $\mathbb{Z}$ is a matrix of size $S \times N$, and each term

$$z^{(1)}_{ij} = \theta^{(1)}_{0i} + \theta^{(1)}_{1i} x_{j1} + \theta^{(1)}_{2i} x_{j2} + \ldots + \theta^{(1)}_{Mi} x_{jM} \tag{4}$$

is the affine transformation of input example j projected onto neuron i of the first hidden layer. In this work, the hidden neurons were defined as rectified linear units (*ReLU*), which means that the activation matrix of the first hidden layer ($\mathbb{A}^{(1)}$) is defined by the element-wise operation:

$$a_{ij}^{(1)} = ReLU(z_{ij}^{(1)}) \triangleq max\{0, z_{ij}^{(1)}\}, \quad \forall i \in [1, S], j \in [1, N]. \tag{5}$$

Then, a row of ones (a bias term for each example i) is added on top of the activation matrix and this new matrix ($\mathbb{A}_b^{(1)}$, of size $(S+1) \times N$) is used to calculate the activations of the second hidden layer using another weight matrix ($\Theta^{(2)}$) such that:

$$\mathbb{A}^{(2)} = ReLU(\mathbb{Z}^{(2)}) = ReLU((\Theta^{(2)})^T \cdot \mathbb{A}_b^{(1)}) \tag{6}$$

and this is repeated for all the hidden layers in the network. It has previously been shown that varying the number of hidden neurons in the layers gives no advantage in the performance of a dense network. Consequently, it is common practice to maintain the number of hidden neurons constant across all the hidden layers. Then, all the matrices $\Theta^{(i)}$ for $i \in [2, L]$, where L is the number of hidden layers in the network, will be of size $(S+1) \times S$ and all the activation matrices $\mathbb{A}^{(i)}$ for $i \in [2, L]$ will be of size $S \times N$.

The output layer of the network is composed by O output neurons. To calculate the output of the network, the activations from the last hidden layer are projected onto the output layer through an affine transformation with a weight matrix of the form:

$$\Theta^{(L+1)} = \begin{pmatrix} \theta_{01}^{(L+1)} & \theta_{02}^{(L+1)} & \dots & \theta_{0O}^{(L+1)} \\ \theta_{11}^{(L+1)} & \theta_{12}^{(L+1)} & \dots & \theta_{1O}^{(L+1)} \\ \theta_{21}^{(L+1)} & \theta_{22}^{(L+1)} & \dots & \theta_{2O}^{(L+1)} \\ \vdots & \vdots & & \vdots \\ \theta_{S1}^{(L+1)} & \theta_{S2}^{(L+1)} & \dots & \theta_{SO}^{(L+1)} \end{pmatrix}, \tag{7}$$

and the output matrix of the network ($\mathbb{O}$) is calculated using a sigmoid activation function for the output units so that:

$$\mathbb{O} = \sigma\left(\mathbb{Z}^{(L+1)}\right) = \sigma\left((\Theta^{(L+1)})^T \cdot \mathbb{A}_b^{(L)}\right) \tag{8}$$

where the element-wise sigmoid function (σ) is defined as:

$$o_{ij} = \sigma(z_{ij}^{(L+1)}) = \frac{1}{1 + e^{-z_{ij}^{(L+1)}}}, \quad \forall i \in [1, O], j \in [1, N]. \tag{9}$$

With these definitions, the output matrix ($\mathbb{O}$) will be of size $O \times N$, where each column corresponds to the output of the network for each of the training inputs, respectively.

Once all the examples have been propagated through the network, it is said that one epoch has passed. At the end of each epoch, the error produced by the network is calculated and a learning step is taken as explained in the following section.

Cost function and gradient descent

In this work, the cost function used was the binary cross-entropy. This is a computationally efficient formulation of the cross-entropy maximum likelihood estimator in the particular case of binary classification. When using one-hot encoding this function can be extended to multi-class classification by considering each output neuron as one binary classifier. Additionally, the function was regularized with weight decay, which prevents the network weights from being too large and, consequently, avoids overfitting the training data. This cost function, calculated at the end of each training epoch, is defined as follows:

$$J(\Theta) = -\frac{1}{M}\left(\sum_{i=1}^{N}\sum_{j=1}^{O} y_{ij}\ln(o_{ji}(\Theta)) + (1 - y_{ij})\ln(o_{ji}(\Theta))\right) + \frac{1}{2M}\sum_{i}\theta_i \tag{10}$$

where Θ is a vector containing all the weights used in the network.

At the end of each epoch, this cost function was calculated and its derivative with respect to each of the weights ($\nabla_\Theta J$) was obtained using the back-propagation algorithm. Then, the weights were updated following the gradient descent:

$$\theta_i \leftarrow \theta_i - \varepsilon\frac{\partial J}{\partial \theta_i}, \tag{11}$$

where ε is the learning rate, and the updated weights were used in the next epoch. This procedure was repeated until the cost was low ($J < 10^{-4}$), there was a small difference in the cost between epochs ($\Delta J < 10^{-10}$) or for a limit of 50000 epochs. The learning rate (ε) needed for the training of each network was obtained by analyzing the training error as presented in Fig. 1. It shows the progression of training error for ANN_1 with 5 hidden layers and 7 hidden neurons per layer. The four training procedures were made using the same set of randomly selected examples and starting with the same randomly initialized weights. Figure 1(A) exemplifies how high learning rates ($\varepsilon = 0.1$ and $\varepsilon = 0.01$) resulted in an oscillating behavior that consistently missed the local minimum of the error, whereas low values ($\varepsilon = 0.001$) converged too slowly. Hence, the value of $\varepsilon = 0.005$ was the best option for learning ANN_1 (see Fig. 1(B)). An equivalent approach was taken to select the learning rates required for ANN_{21} ($\varepsilon = 0.01$) and ANN_{22} ($\varepsilon = 0.1$).

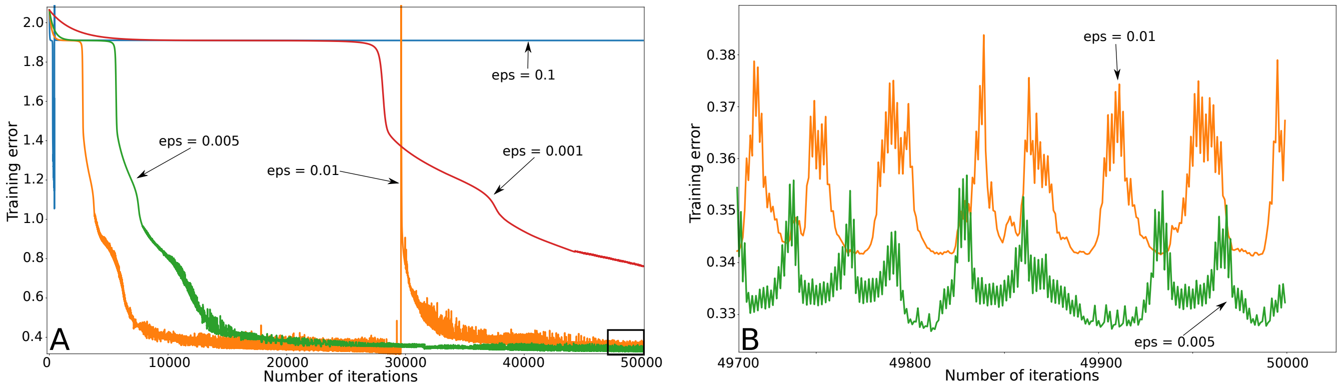

Fig 1. Training error as a function of the number of iterations for different learning rates (eps). (A) Training error calculated in 50000 gradient descent iterations, (B) Detail of the last 300 iterations (i.e. Bottom right corner of (A)).

10-fold cross-validation

It is clear that using all the examples from the database for training would produce overfitting. Moreover, a way of estimating the generalization error of the models is required. Consequently, each neural network reported in this paper was trained using a 10-fold cross-validation as follows.

First, all the examples were divided into a training set ($\mathbb{X}_{train}$) and an evaluation set ($\mathbb{X}_{eval}$), which contained 75% and 25% of the data, respectively. The examples to be included in each subset were selected randomly, using a uniform distribution, which preserved the balance in the classes. The evaluation set is not used during training, so the performance of a model in this set can be regarded as an estimate of its generalization capability.

Second, the training set was divided into ten subsets of equal size (i.e. $\mathbb{X}^{(1)}_{train}$, $\mathbb{X}^{(2)}_{train}$, $\ldots$, $\mathbb{X}^{(10)}_{train}$). As before, these were selected randomly following a uniform distribution. The deep learning model was then trained, as explained before, using the combination of sets $\mathbb{X}^{(2)}_{train}$ until $\mathbb{X}^{(10)}_{train}$ and, once trained, its F_1-score was calculated on $\mathbb{X}^{(1)}_{train}$. Subsequently, the training was repeated but leaving $\mathbb{X}^{(2)}_{train}$ out for testing, then $\mathbb{X}^{(3)}_{train}$, and so on until all sets were used for testing. Finally, the model that produced the highest F_1-score was selected as the best model and it was applied on $\mathbb{X}_{eval}$.

The sensitivity, positive predictive value and F_1-score reported in this work are those obtained when applying to the evaluation set the model that performed best during the cross-validation . It should be observed that dividing the database into a training and an evaluation set enables the estimation of the generalization error of the trained model because it is tested in data that was not used during training. Furthermore, training using cross-validation allows to avoid many of the common issues found in machine learning such as overfitting and bias and helps minimizing the effect of outliers in the data.

Baseline performance

From the results shown in the main text, it should be evident that this classification task is not trivial. Indeed, the overlap in the values of the features measured from the pECGs imply that ischemia can manifest itself in different pECG morphologies. However, in order to provide a baseline performance to compare to, three logistic regression classifiers were trained. Namely, the output of a logistic regression classifier is:

$$o = \sigma(\theta_0 + \theta_1 x_1 + \theta_2 x_2 + \dots \theta_M x_M), \tag{12}$$

and the weights (θ_i) can be trained following the same learning procedure and cross-validation explained before. Three logistic classifiers were trained, which used as inputs the magnitude of the pECG features: LR_1 classified between *Control* and any other signal, LR_2 between *Mild* and any other signal and LR_3 classified between *Severe* and any other signal. It should be observed that, in this learning task, there is a slight misbalance between the classes; however, this is not expected to have a significant impact in the performance of the model. The evaluation metrics obtained after training these models are presented in Table 1.

Table 1. Performance of the logistic regression classifiers.

Classifier	Se (%)	PPV (%)	F_1-score (%)
LR_1	97.87	63.72	77.37
LR_2	95.37	56.33	70.83
LR_3	99.67	92.49	95.95

Deep learning performance

The optimal results presented in the main text were obtained through a grid search approach. Namely, for each of the models proposed (i.e. ANN_1, ANN_2 and ANN_3), the number of hidden layers (L) and hidden neurons per layer (S) were varied incrementally, starting at 1, until an optimal performance was obtained. The results of most of the experiments made are presented in Tables 2, 3 and 4, for ANN_1, ANN_2 and ANN_3, respectively. For succinctness, the network topologies that were uncapable of learning or had a performance worse than the linear regression classifiers are not shown in the tables. The optimal topology was considered found once no significant improvement was observed by incresing the complexity of the network or when a decrease in performance was observed. The tables highlight in bold the topology that showed the best performance and, consequently, was reported in the main text.

Table 2. Performance of all neural network topologies for ANN_1.

L	S	Se (%)	PPV (%)	F_1-score (%)
4	7	91.67	91.46	91.56
4	8	94.56	94.24	94.39
4	9	92.77	92.46	92.62
4	10	93.88	93.99	93.94
**5**	**7**	**94.78**	**95.52**	**95.15**
5	10	92.56	92.56	92.56
5	15	92.00	92.20	92.10
5	20	93.00	92.90	92.95
5	25	92.44	92.55	92.50
6	7	93.22	93.43	93.33
6	10	93.00	93.10	93.05
6	15	93.00	92.90	92.95
6	20	93.89	93.99	93.94
6	25	94.00	94.21	94.10
7	10	92.00	92.00	92.00
7	15	94.44	95.08	94.76
7	20	91.22	91.83	91.53
7	25	93.67	93.77	93.72

Finally, to give an idea of the cross-validation training, testing and evaluation process, Table 5 shows the performance of the best models (highlighted in the previous tables) in the best cross-validation fold. The table shows the sensitivity, positive predictive value and F_1-score obtained at the end of training and the previously reported evaluation performance.

Table 3. Performance of all neural network topologies for $\mathbf{ANN_{21}}$.

L	S	Se (%)	PPV (%)	F_1-score (%)
3	7	96.46	96.14	96.30
3	8	94.80	97.25	96.01
3	9	97.34	96.07	96.70
3	10	94.76	95.98	95.37
3	11	94.99	96.28	95.63
3	12	95.88	96.68	96.28
4	7	94.00	94.65	94.32
4	8	96.44	93.43	94.91
4	9	97.29	94.10	95.67
4	10	95.70	95.54	95.62
4	11	98.61	90.43	94.34
4	12	94.82	95.95	95.38
5	7	94.49	96.26	95.37
**5**	**8**	**99.00**	**96.28**	**97.62**
5	9	93.81	97.06	95.41
5	10	97.31	93.54	95.39
5	11	97.31	94.61	95.94
5	12	98.83	92.90	95.77

Table 4. Performance of all neural network topologies for $\mathbf{ANN_{22}}$.

L	S	Se (%)	PPV (%)	F_1-score (%)
3	3	99.68	99.68	99.68
3	5	99.32	99.32	99.32
3	6	99.68	99.35	99.52
4	3	100.00	99.67	99.84
**4**	**4**	**100.00**	**100.00**	**100.00**
4	5	100.00	100.00	100.00
4	6	100.00	100.00	100.00

Table 5. Training, testing and evaluation performances of the best classifiers found.

Classifier	Training			Evaluation		
	Se	PPV	F1	Se	PPV	F1
ANN_1	94.44	94.42	94.43	94.78	95.52	95.15
ANN_{21}	99.75	90.82	95.08	99.00	96.28	97.62
ANN_{22}	98.96	98.98	98.96	100.00	100.00	100.00
