# Supplementary material for: A modeling and machine learning approach to ECG feature engineering for the detection of ischemia using pseudo-ECG
Source: PLoS One. 2019 Aug 12;14(8):e0220294. doi: 10.1371/journal.pone.0220294 (PMC6690680; doi:10.1371/journal.pone.0220294)
Supplement: S2 File — Contains the figures that complement the presented results and further support the discussion. (PDF) [file pone.0220294.s002.pdf]

Supplementary figures

The figures provided in this section complement the results presented in the main text and give further support to the arguments given in the discussion.

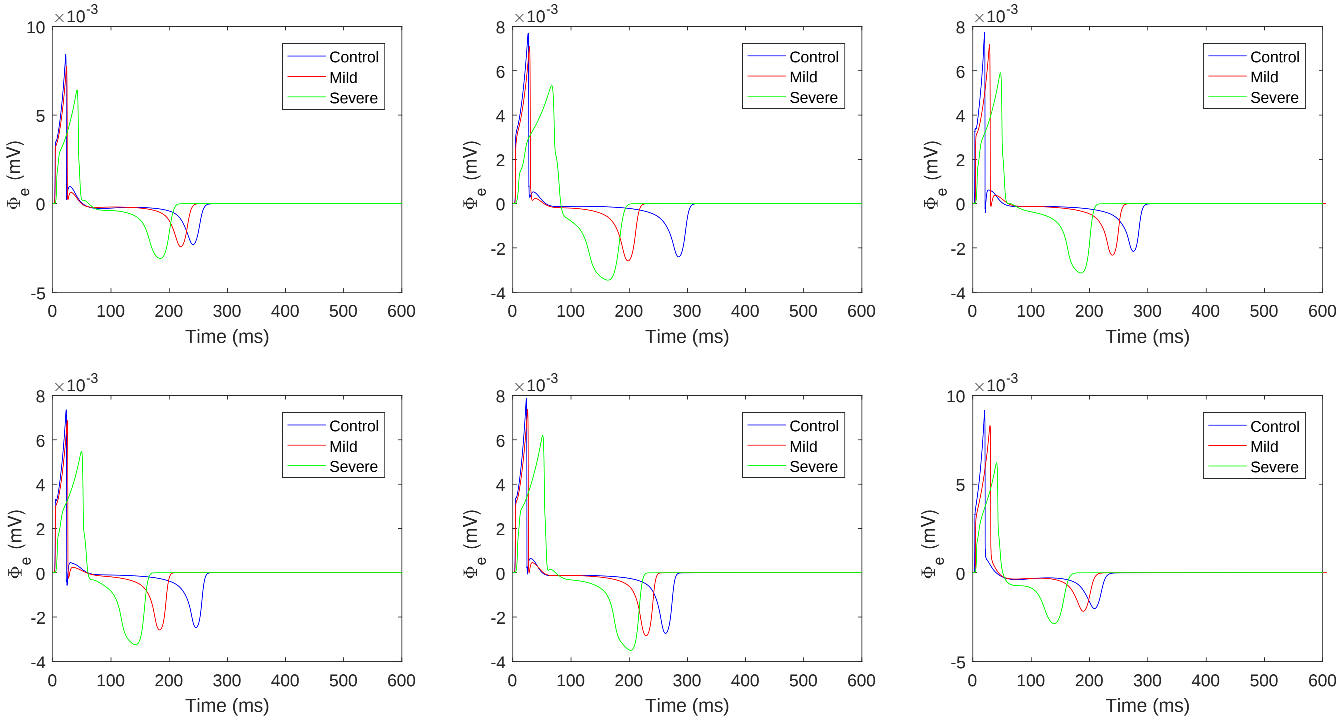

Fig 1. Representative examples of pECG calculated from TP06 cables of cells. Each figure presents the three pECGs calculated with a single model using the Control, Mild and Severe ischemic parameters. These correspond to the simulations made with a full ischemic cable. The traces obtained for the ORd cables were largely similar

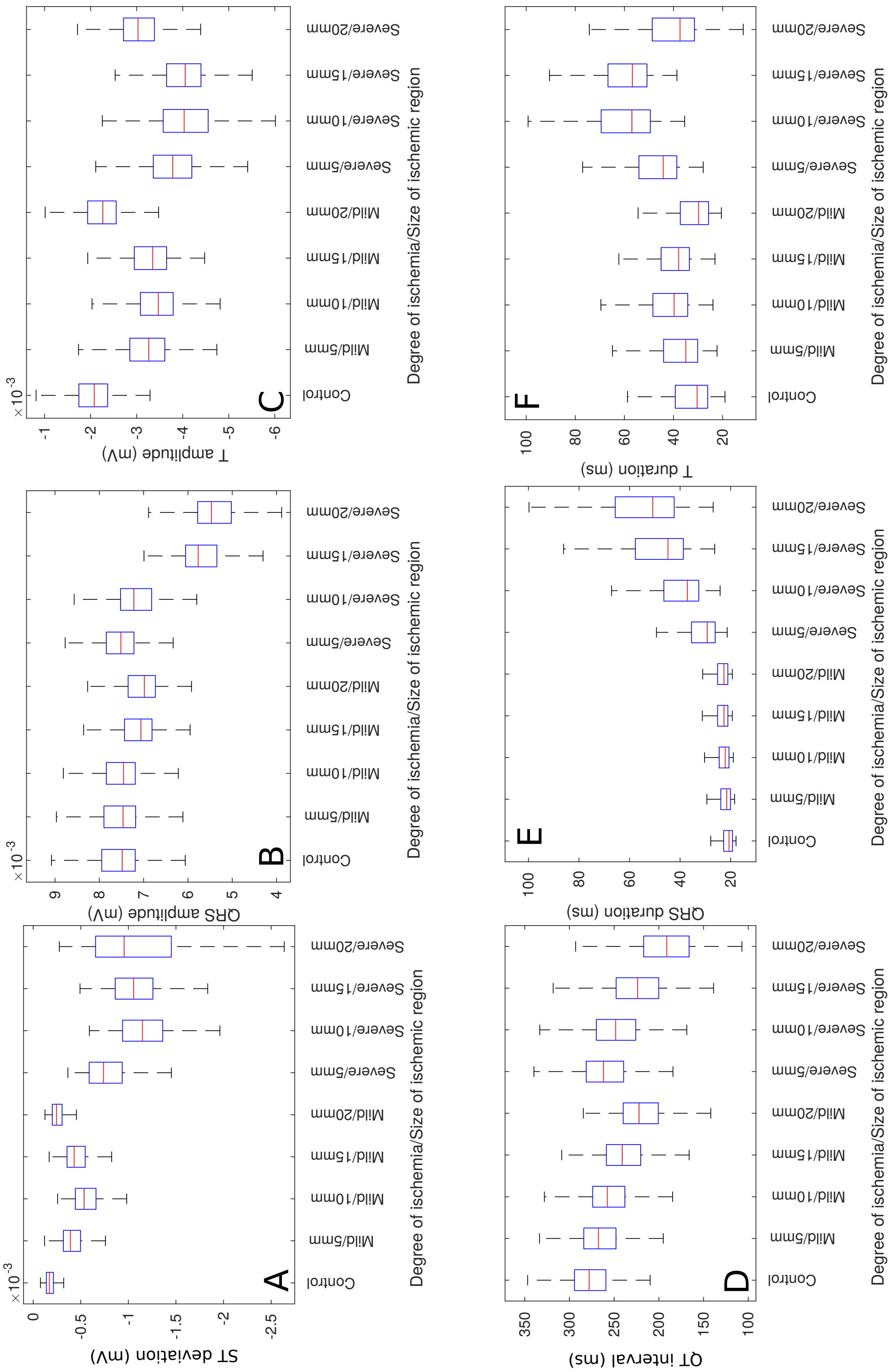

Fig 2. Pseudo-ECG biomarkers of TP06 cables of cells as a function of the size of the ischemic region. (A) ST deviation, (B) QRS amplitude, (C) T wave amplitude, (D) QT interval, (E) QRS complex duration, (F) T wave duration.

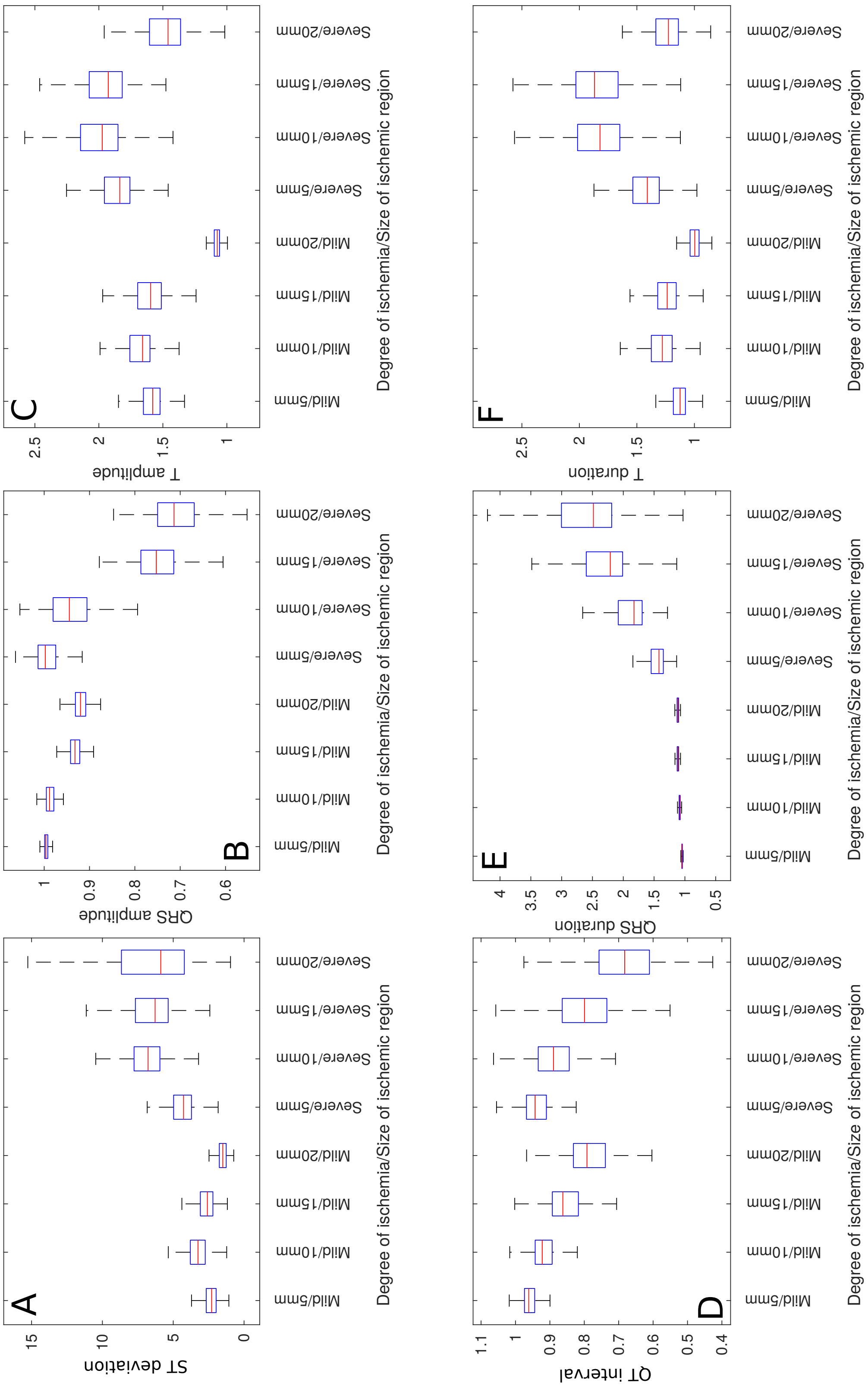

Fig 3. Ratio of change of pseudo-ECG biomarkers of TP06 cables of cells as a function of the size of the ischemic region. (A) ST deviation, (B) QRS amplitude, (C) T wave amplitude, (D) QT interval, (E) QRS complex duration, (F) T wave duration.
